# Smoked cocaine in socially-depressed areas

**DOI:** 10.1186/1477-7517-7-27

**Published:** 2010-11-09

**Authors:** Jordi Delas, Elena Adán, Olga Díaz, Margarita Aguas, Montserrat Pons, Ricardo Fuertes

**Affiliations:** 1 SAPS Creu Roja. (Av. Drassanes 13-15), Barcelona 08001, Spain; 2 Departamento de Medicina, Universitat de Barcelona. (Casanova, 143), Barcelona 08036, Spain; 3 Hospital Universitari del Sagrat Cor. (Viladomat, 288), Barcelona 08029, Spain

## Abstract

**Background:**

The main objectives of this study are to describe the smoked cocaine user's profile in socially-depressed areas and their needs from a harm-reduction perspective, to investigate their use of smoking crack and compare the acute effects between injecting and smoking consumption.

**Methods:**

The study took place in SAPS, Barcelona, Spain. Two focus group sessions were undertaken with a total of 8 drug users. Secondly, the 8 participants answered a structured questionnaire and in the course of the sessions, as a snowball activity, were trained to survey 6 other crack smokers.

**Results:**

We obtained 56 questionnaires. The majority of participants were from non-European Community countries (62.69%), 70.2% of participants referred to sharing the smoking equipment. The most frequent symptoms reported during smoked cocaine were mydriasis (83.33%)), perspiration (72.92%) and compulsive object search (70.83%) During the group sessions, participants said that smoked cocaine is much more addictive than injected cocaine and causes more anxiety. Participants also reported the difficulty of changing from injected use to smoked use, due to the larger amount of cocaine needed to reach the same effects as when having injected.

**Conclusions:**

We can conclude that the research, focused on achieving greater knowledge of the smoked cocaine user's profile, their usage of smoking crack, consumption patterns and acute effects, should be incorporated into substance misuse interventions.

## Background

SAPS Creu Roja is a harm-reduction center located in Ciutat Vella, one of the main areas of drug use and dealing in Barcelona, Spain. It provides health and social care, and psychological and legal assistance to drug users in socially-deprived situations. 86% of the people attended to are men, 13% women and 1% transsexuals, and 85% are homeless. Opened in Barcelona in 1993, it is located in one of the main drug use and dealing areas in Barcelona. Until the end of December 2008 the center received 58,978 visits and since the beginning 11,381 different people have been registered. In 2008, 811 people were attended to for the first time and on average 154 different people visit the service per day. During 2008 the index of syringes recovered was 0.35, with 90,488 syringes delivered and 32,104 recovered. The human resources are 1 coordinator, 4 nurses, 6 social educators, 1 medical internist, 1 lawyer and 1 psychologist. Condoms, new needles and smoking equipment are also offered for free.

In 2003, a drug consumption room for injected use, supervised by healthcare professionals, was added to the initial needle exchange program. This drug consumption room allows the professionals to directly observe the injected heroin and cocaine consumption and their acute effects [1] and act in emergency situations.

Like many harm-reduction programs in the southern Europe, SAPS was primarily focused on injected drug use [2]. The change in the method of consumption from injecting to smoking is recommended to reduce the blood-borne infectious diseases such HIV, Hepatitis B and C, and other diseases directly related to injection, such as abscess, phlebitis or thrombosis. It is also possible to reduce mortality related to acute reactions to intravenous consumption [3].

However, merely suggesting is not sufficient to promote this change of method of consumption from injecting to smoking. We must have an in-depth knowledge of the specific characteristics of smoked use and that the harm-reduction centres can provide the chance to smoke instead of injecting. In many cities crack smokers have been largely ignored in the development and implementation of harm-reduction programmes. [4,5]

The main objectives of this study are to describe the smoked cocaine user's profile in socially-depressed areas and their needs from a harm-reduction perspective, to investigate their use of smoking crack and consumption patterns and compare the acute effects between injecting and smoking consumption.

## Methods

**The study took place **in SAPS Creu Roja, Barcelona, Spain and was included in the context of the workshops organized annually by the Program on Substance Abuse of the Ministry of Health of Catalonia, based on snowball activities [6].

A twofold methodology was used:

### 1. Focus groups

On the one hand, two focus group sessions of two hours each were undertaken with a total of 8 drug users. Participants were recruited in January 2008, firstly from those who attended our centre, according to the following criteria: they have smoked cocaine and have minimum language skills in speaking, reading and writing. The sessions were managed by three staff members (two nurses and one social worker) following a semi-structured script. The lines of discussion were the health risk of smoking drugs, mainly using crack, smoking equipment and the transmission of infectious diseases. Participants were asked to make pipes with the material they use and discuss its specific risks. Also discussed was a comparison of both ways of consumption, injecting and smoking, by presenting the most frequent effects observed in our drug-consumption room.^1 ^Also discussed was the acceptance and need for harm-reduction interventions such as safe smoking equipment and drug consumption rooms for smoking users. In this focus group, the data was collected through field notes of the three staff members, who at the end of the session compared their notes.

### 2. Structured questionnaires

Secondly, the 8 participants of the focus groups answered a structured questionnaire and in the course of the sessions, as a snowball activity, were trained to survey 6 other crack smokers who were not clients of our centre. The questionnaire includes some questions in the first part that always appear in the enquiries organized annually by the Program on Substance Abuse of the Ministry of Health of Catalonia, based on snowball sampling. A second specific group of questions was designed specifically for this piece of research, taking into account current socio-demographic data, health information (respiratory diseases and treatment), history of drug use, characteristics of smoked cocaine (material for cooking from hydrochloride to base cocaine, kinds of handmade pipes, patterns of use) and about their acceptance of the possibility of attending a drug consumption room for smoking. The last part of the questionnaire was a list of the effects of injected cocaine observed in our drug consumption room ^1 ^and participants were asked to answer "yes or no" if they notice when smoking.

Descriptive statistics were used to determine variable frequencies for all items of the questionnaire.

### Commitment contract and informed consent

Confidentiality measures: the focus group's participants signed an informed consent authorising the researchers to use their data. They also signed a commitment contract through which they were going to respect the confidentiality of their respondents.

### Control of quality and validity of questionnaires

Within the interviewers' training, communication skills were assessed using a standard questionnaire designed by the Program on Substance Abuse of the Ministry of Health of Catalonia. Participants were asked for difficulties and possible obstacles to doing the survey. These were also assessed once after the questionnaires were completed and returned to the researchers.

The 8 main participants were paid €40 for their participation in the sessions and €10 more for each survey administered by them. All participants were guaranteed that any information they provided would remain strictly anonymous and confidential. In the same contract mentioned above, the participant was informed that their active participation, and the quality and validity of the data given to the researchers was essential to be paid for it. These aspects were evaluated by the research team analysing the questionnaires.

The study was submitted to the Program on Substance Abuse of the Ministry of Health of Catalonia where it was assessed in terms of ethics and validity. Data was processed using the SPSS 15 statistical program.

## Results

### 1. Structured questionnaire

We obtained 56 questionnaires, 8 from participants in the group sessions and 48 from the snowball sample.

1. Socio-demographic data showed that 80% were male (n = 45) with an average of age of 32.6 (range from 19 to 63 years old). The majority of participants were from non-European Community countries (62.69%). 45.5% stated they did not have identity documents and 38.2% did not have a health card.

Responding to where they live, 22 (39.3%) were sleeping rough and 14 (25%) were squatters. Regarding their main financial income, 46.3% stated petty crime, 7.40% were receiving financial benefits and only 3.70% had a temporary job (Table 1).

**Table 1:** Interviews: Socio-demographic data.

Gender	Men: 45 (80%)	Women: 11 (20%)					
Average age		32.68 ± 3.74 years old (confidence interval 95%) (range of: 19 to 63 years old)					
Country of origin		Spain:			11		19.6%
		Other European Community countries:			21		42.8%
		Non-European Community countries:			24		37.6%
Average time living in Spain (not born in Spain)				6.13 years (range of 0.16-28 years)			
Identity Document				YES: 30 (54.4%)	NO: 25 (45.5%)	MISSING: 1 (1.8%)	
Health card				YES: 29 (51.8%)	NO: 27 (48.2%)		
Using soup kitchens and charity settings				17 33.30%			
Housing	Sleeping Rough	22	39.3%	Main Income	Petty crime	25	46.3%
	Squatters	14	25%		Financial aid	4	7.4%
	Own house	8	14.3%		Temporary job	2	3.7%
	Hostel	3	5.4%		Missing	2	3,6%
	Others	9	16%		Others	22	42.7%

Relating to drug use, at the time of the interview only one person was in withdrawal and 5 were not current crack cocaine smokers. Of those who were cocaine smokers in that moment (n = 42), 19 (45.23%) only smoked and 23 (54.76%) combined smoking with snorting or injecting. Of those 42 current cocaine smokers, 39 (92.85%) were using this drug in combination with some kind of depressants such as methadone, heroine, benzodiazepines or alcohol.

When participants were asked about some characteristics of smoking crack cocaine, 76.8% responded that they preferred to use ammonia to convert the cocaine hydrochloride into cocaine base or crack, compared to 41.1% who preferred sodium bicarbonate. The paraphernalia used were handmade pipes in 87.5% of responses, 76.4% usually smoked with someone else and 70.2% of participants referred to sharing the smoking equipment.

3) Regarding the place of consumption, 48.10% smoked cocaine in public places, 29.60% in private places, whereas 22.20% referred to doing it in both (Table 2). 44.4% of users interviewed knew about existing consumption rooms for smokers in other countries. 72.7% considered them as necessary and 65.1% would use them if they were available (Table 3).

**Table 2:** Interviews: Drug use data.

	Cocaine	Heroine	Alcohol	Benzodiazepines	Cannabis
**Drug use**	47 (83.9%)	44 (78.6%)	30 (53.6%)	15 (26.8%)	35 (62.5%)
	Cocaine, heroine, alcohol, benzodiazepines and cannabis			6	10.7%
**Drugs alone or**	Cocaine, heroine, alcohol and benzodiazepines			9	16.1%
**combined**	Cocaine, heroine and alcohol.			26	46.4%
	Cocaine and heroine			40	71.4%
	Cocaine			47	83.9%
**SMOKED COCAINE**					
Crack cocaine preparation	Ammonia 43 (76.8%)		Baking soda 23 (41.1%)		
Smoking equipment	Bottle Pipe 49 (87.5%)		Foil 25 (44.6%)		
Smoke pipes	Yes 47 (83,9)		Share pipes (% of those who smoke pipes) 33 (70.2%)		
Alone or with someone else	Alone 7 (12.7%)		Someone else 42 (76.4%)	Both 6 (10.9%)	Missing 1
Where do they smoke?	Public place 26 (48.10%)		Private place 16 (29.60%)		At home 16 (29,6)
**Daily average dose (grams per day)**					
**N**	**%**		**Snorted**	**Smoked**	**Injected**
18	32.14		1.32 g/day	3.4 g/day	-----
8	14.28		1.29 g/day	-----	2.58 g/day
14	25.00		----	4.45 g/day	2.02 g/day

**Table 3:** Cocaine effects.

EFFECTS	% INJECTED	% SMOKED
Mydriasis (pupil dilation)	29.8	83.33
Perspiration	35.1	72.92
Object Search	14.9	70.83
Verbosity	49.4	66.67
Tachypnea (respiratory rate rise)	1.8	64.58
Compulsive cleaning and tidying	1.8	62.5
Tactile hallucinations	3.0	60.42
Agitation	9.5	58.33
Auditory hallucinations	8.9	58.33
Salivation	14.9	56.25
Persecution complex	3.6	54.17
Confusion	7.1	52.08
Repeated Movements	9.5	50
Tachycardia	8.3	50
Chest pain	0	45.83
Visual hallucinations	13.7	35.42
Temporary paralysis	5.4	33.33
Convulsions	0	31.25
Delirium	1.2	29.17

4) The most frequent symptoms reported during smoked cocaine were mydriasis (83.33%)), perspiration (72.92%) and compulsive object search (70.83%) (Table 3).

### 2. Focus group

During the group sessions we were able to collect information from the discussion sessions on issues such as: substances, their effects and the method of smoking crack cocaine. We also asked about comparing smoked and injected use.

Participants said that smoked cocaine is much more addictive than injected cocaine and causes more anxiety. Some of them find this method of consumption unpleasant.

Talking about the health risks of smoking crack, participants were sceptical about the idea that infectious diseases such as HIV or HCV can be transmitted through pipes, so they usually share smoking equipment. Nevertheless many users were more familiar with other diseases such as tuberculosis or other pulmonary diseases. Other problems that they easily related to smoking crack were cuts and burns on hands and lips due to inadequate material for making the pipes.

Participants were asked to make pipes in a session using material that they usually have. The most common objects were plastic water or methadone bottles. The top is replaced by a piece of foil fixed with an elastic (usually a part of a latex glove or a condom), which is perforated with a needle. A hole is made in the side of the bottle, usually burning the plastic with a cigarette, where a piece of a syringe is placed and used as a stem. Other materials used are plastic glasses and medical inhalers. Smoking on foil is not very common when using crack, participants explaining that it is used when smoking heroin or both substances at the same time.

When referring to the substances used to convert the cocaine hydrochloride to cocaine base participants explained that using ammonia is easier than sodium bicarbonate. It consists of mixing the ammonia with powdered cocaine, then an oily drop is obtained that can be separated and when dry becomes a rock. This makes it easier when the consumption is in the street or when the user has not too much experience. Another reason to use ammonia instead of sodium bicarbonate was the flavour it has when cocaine is smoked. Smoking cocaine, like injected cocaine, is usually combined with the use of depressants such as heroin, benzodiazepines or alcohol, which reduces the strong effects of cocaine. Those participants using other methods of consumption described smoked cocaine as more compulsive as it generates more anxiety, and some of them even found it unpleasant.

They explained that the most frequently combined methods of consuming are to snort and smoke, but the injected use is not usually combined with any other method due to the larger amount needed to reach the same effects. Participants also reported the difficulty of changing from injected use to smoked use, due to the larger amount of cocaine needed to reach the same effects as when having injected. [7] Due to the cost and availability of crack cocaine, they reported preferring to buy powdered cocaine and cook it by themselves to making crack cocaine (in their environment 50 mg of cocaine costs €5, whereas 200 mg of powdered cocaine costs €10). The majority reported sharing pipes as they use them to smoke with friends or acquaintances, but not with strangers.

Their opinion about smoking rooms is controversial (Table 4). They said that the acute effects of smoked cocaine, such as agitation and aggressive behaviour, would require a lot of control from professionals, as well as protection and security. They mentioned the existence of what are called "Crack Houses", where drug dealers and drug users buy, sell, produce, and use illegal drugs, including, but not limited to, crack cocaine. However, these are found in some places in Europe and the USA, but not in Spain.

**Table 4:** Interviews: Their opinion about drug consumption rooms

DRUG CONSUMPTION ROOMS	n	%
**Know about them**	24	44.4%
**Consider them needed**	40	72.7%
**They would use them**	28	65.1%

## Discussion

Harm-reduction brings hidden marginalised drug users in contact with social and health services. Crack smoking involves particular risks and harms, which underlines the need for targeted interventions [8]. In this study, the snowballing methodology enabled us to be in contact with cocaine smokers who were not attending the harm-reduction centres and provided us with very important information [9]. The primary goal of most harm-reduction approaches is to meet individuals "where they are at" [10]. Despite this method being known to have limitations and bias, in hidden populations it is currently still one of the slightly better methods [6].

Enquiries in which external pollsters to the researcher group are needed represent a weak point. However, in illegal areas of study it is even more complicated. Some groups' researchers use a snowballing method characterized by assigning names to people who will be contacted. It was also complicated in this study because the aim was to interview people who do not have contact with our service [6]. This kind of survey is not practical for researching strictly defined questions or attributing causal relations. But when the aim is to share information based on scientific arguments about drug users who smoke cocaine, this methodology continues to have an important role. It involves sharing hot and cold information between reality and scientific concepts. The 8 main participants were paid. This provided an incentive to them to complete the surveys and also gave them recognition or respect for their role and skills.

The analysis of demographic characteristics shows that the participants of our study are in a socially-deprived situation according to one of our objectives, to find out more about their condition and to represent the current profile of illegal drug users: men, foreigners, without access to basic needs (lodging, food, hygiene and healthcare) [11,12].

Survey results show that 48.10% smoke in public. This may have a negative impact on the community, and can also aggravate the risk for users, due to the difficulties in preparing the substance properly and environmental conditions of consumption [13,14]. Implementation of specific harm-reduction strategies for crack smokers are necessary among this population, focusing on social and health care as well as strategies directly related to safety, including on-site preparation of the cocaine to be smoked.

Results show that the use of pipes is important for 70% and in this case the rate of shared pipes is 70.2%. Supervised inhalation and smoking sites could increase the availability of new pipes, mouthpieces for pipes and education for safer consumption. Smoking of crack cocaine was found to be an independent risk factor for HIV seroconversion among people who injected drugs [15]. The authors suggest that wounds in and around the mouth from using metal or glass pipes may make people who smoke crack cocaine more vulnerable to HIV transmission during activities such as oral sex or sharing of crack pipes. As a part of a comprehensive HIV prevention strategy, harm-reduction programs should address the unique needs of people who smoke crack cocaine [16].

The change by cocaine injectors to smoking crack cocaine may be easier for those who have smoked before, and could be more bearable if they have smoking equipment available^4 ^and a safe and private place to smoke.

More effects were recalled through the discussion sessions when compared with those we observed in the drug-injecting room, perhaps due to the users' expression of their own personal experience, which cannot be discovered entirely through observation only [17,18].

As in the case of injected use, drug-taking rooms make the direct observation of effects possible, which as participants mentioned, could be greater than when cocaine is injected. This fact can be related to the differences in cocaine metabolism in these different ways of administrating the drug [19].

## Conclusions

We can conclude that the research, focused on achieving greater knowledge of the smoked cocaine user's profile, their usage of smoking crack, consumption patterns and acute effects, should be incorporated into substance misuse interventions, disease prevention and health promotion policy in order to provide a better response [20,21].

## Competing interests

The authors declare that they have no competing interests

## Authors' contributions

JD, EA. OD and RF designed the study and wrote the protocol. MA and MP undertook the statistical analysis. JD and EA wrote the first draft of the manuscript. All authors contributed to and have approved the final manuscript.
